# Supplementary material for: A hand-off of DNA between archaeal polymerases allows high-fidelity replication to resume at a discrete intermediate three bases past 8-oxoguanine
Source: Nucleic Acids Res. 2020 Sep 30;48(19):10986–97. doi: 10.1093/nar/gkaa803 (PMC7641752; doi:10.1093/nar/gkaa803)
Supplement: gkaa803_Supplemental_File [file gkaa803_supplemental_file.pdf]

SUPPLEMENTAL DATA

Supplemental Table S1: DNA substrates used in this study

Substrate Primers

Name	Position relative to lesion	Sequence (5'-3')
p18	-5	GCGAGGCGAGCGCGAGCG
p22	-1	GCGAGGCGAGCGCGAGCGATAC
p23	0	GCGAGGCGAGCGCGAGCGATACC
p24	+1	GCGAGGCGAGCGCGAGCGATACCG
p25	+2	GCGAGGCGAGCGCGAGCGATACCGC
p26	+3	GCGAGGCGAGCGCGAGCGATACCGCG
p41	-1	GCGAGGCGAGCGCGAGCGATACCGCGATCGAGTGCAAGCTT
FAM-p22	-1	FAM-GCGAGGCGAGCGCGAGCGATAC
FAM-p26	+3	FAM-GCGAGGCGAGCGCGAGCGATACCGCG

Substrate Templates

Name	Description	Sequence (5'-3')
$t52_{Und}$	Undamaged 52mer template	CGTCCAACATGAAGCTTGCACTCGATCGCGGTATCGCTCGCGCTCGCCTCGC
$t52_{8}$	8-oxo-G template (@23)	CGTCCAACATGAAGCTTGCACTCGATCGC8GTATCGCTCGCGCTCGCCTCGC
$t52_{AP}$	Abasic template (@23)	CGTCCAACATGAAGCTTGCACTCGATCGC_GTATCGCTCGCGCTCGCCTCGC
$t52_{Alt8}$	Double 8-oxo-G template (@23 and 42)	CGTCCAACAT8AAGCTTGCACTCGATCGC8GTATCGCTCGCGCTCGCCTCGC

"8" - 8-oxoguanosine; "_" - abasic site

Cloning Primers

Name	Sequence (5'-3')
SsoPBP1+NdeI FWD	ATTACATATGTCAACGAGATGGCTACCTAAG
SsoPBP1+XhoI REV	ATTACTCGAGTTACTCCTCTTCACTTTCTTCTTCAC
SsoPBP2+NdeI FWD	ATTACATATGAATACTGGATTAATATATCTTATGTCTGTTAATC
SsoPBP2+XhoI REV	ATTACTGCAGTCACTTCTTGTCAGTAGATTTCCTCAC

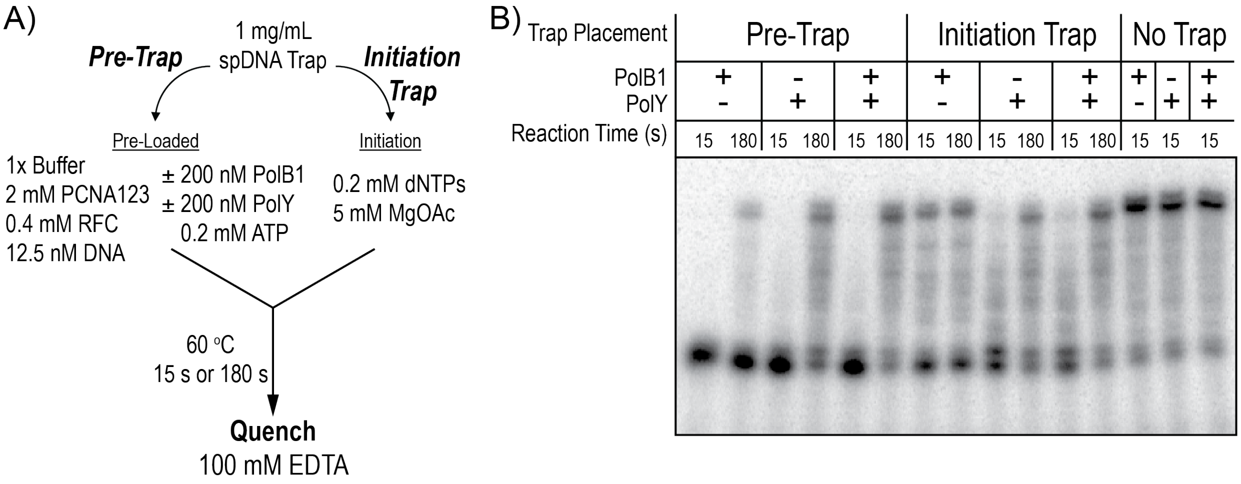

Supplemental Figure S1: Pre-trapping with 1 mg/mL of spDNA still allows for distributive Pol activity. A) 1 mg/mL of spDNA was introduced to the steady-state polymerase assay either by pre-mixing with the indicated Pol complex and undamaged T DNA substrate ('Pre-Trap'), added upon initiation with dNTPs and Mg^{2+} ('Initiation Trap'), or in the absence of trap ('No Trap'), and quenched at indicated time points. B) All 'Pre-Trap' Pol complexes demonstrate time-dependent activity, indicating that this concentration of trap still allows for distributive exchange of Pols from solution through an altered multieuilibria.

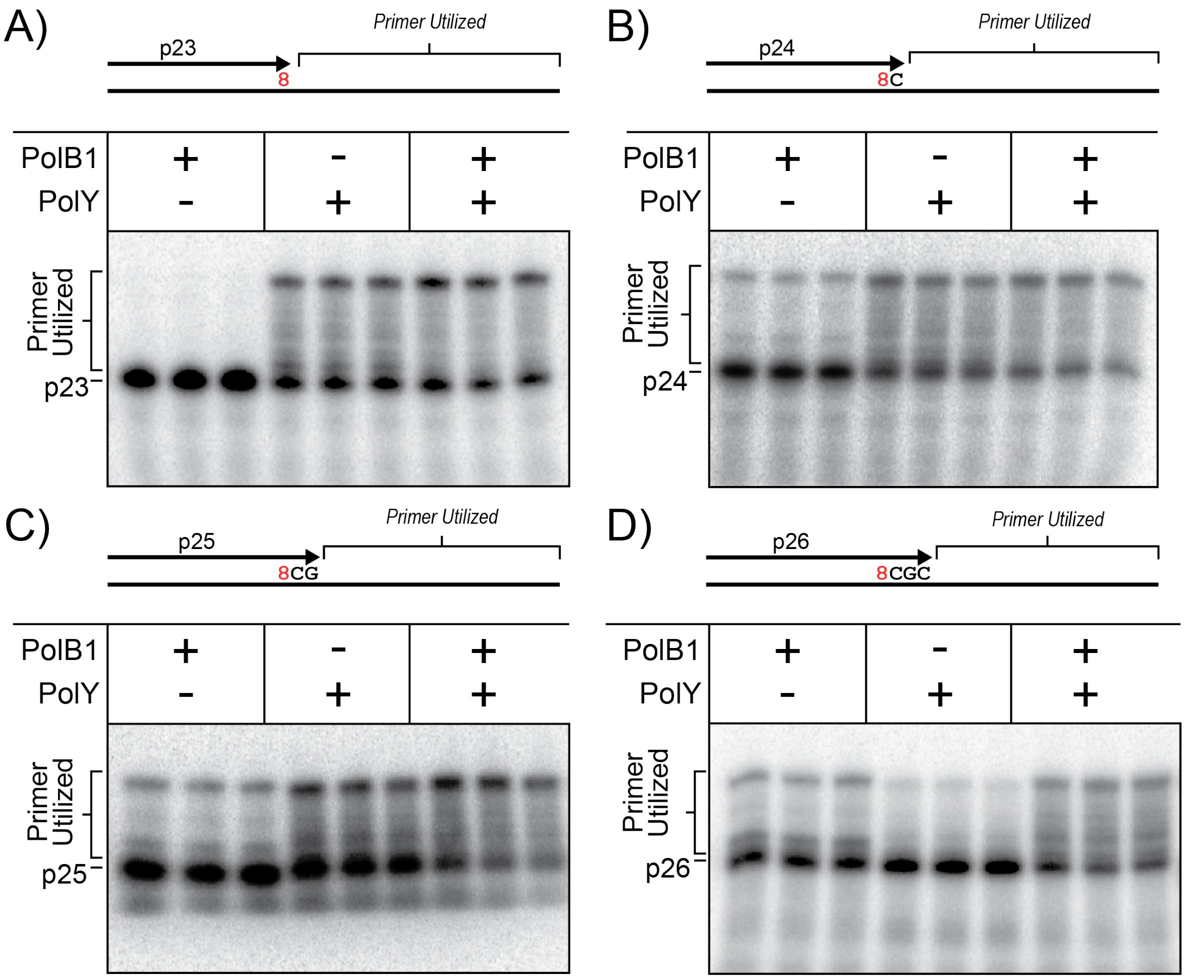

Supplemental Figure S2: Gel images of steady-state lesion bypass by each Pol complex on progressively longer primers. Reactions described in **Figure 1A** were performed on 8-oxoG damaged DNA substrates containing progressively longer primers A) p23, B) p24, C) p25, and D) p26. Products were quantified as a percentage of primer utilized (brackets) and plotted in **Figure 1D**.

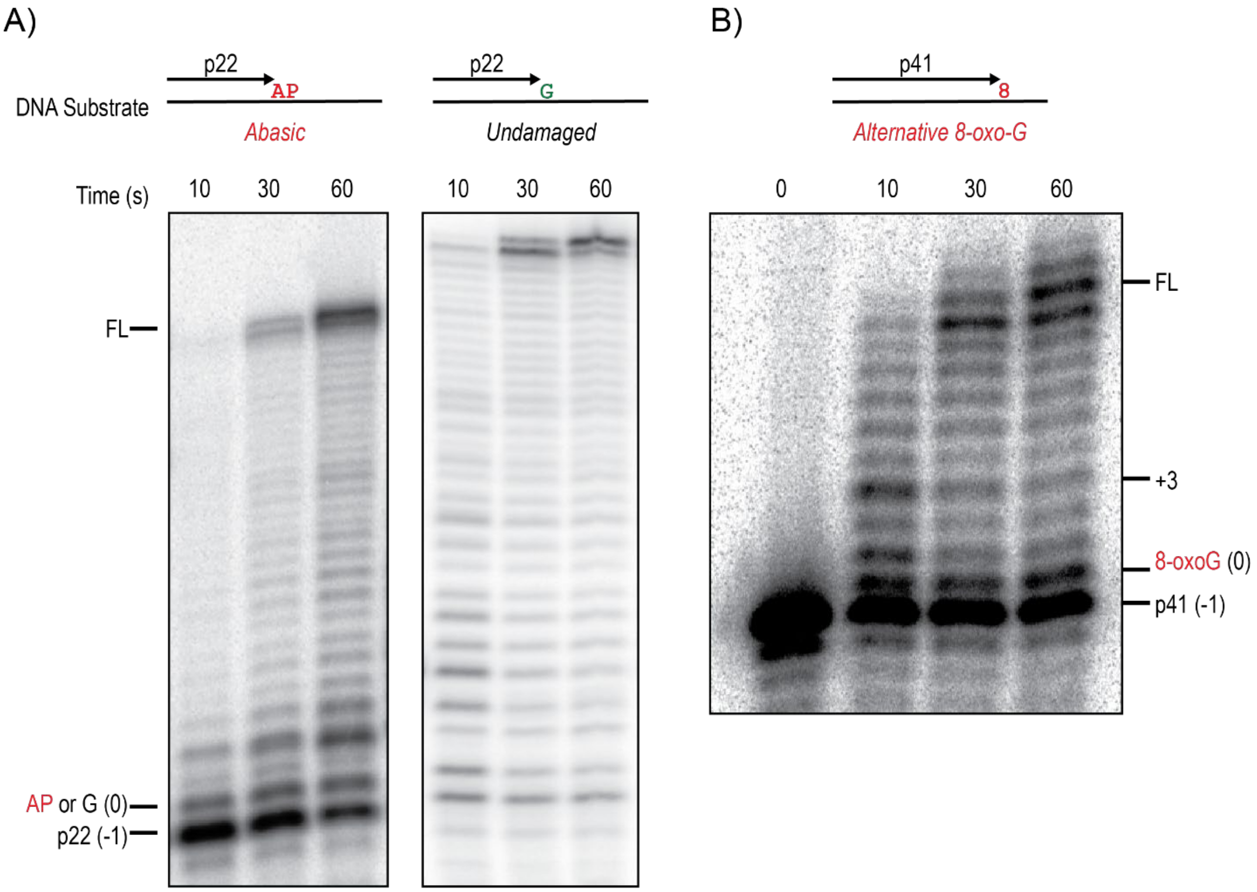

Supplemental Figure S3: The +3 intermediate is characteristic of PolY bypass of 8-oxoG and is not an artifact of sequence context beyond the lesion. Pre-steady-state lesion bypass kinetics reactions were performed by *Scheme i* on DNA substrates containing A) an abasic site (AP) lesion or an undamaged G. Similar reaction were also performed on an alternative 8-oxoG template with a different sequence context beyond the lesion.

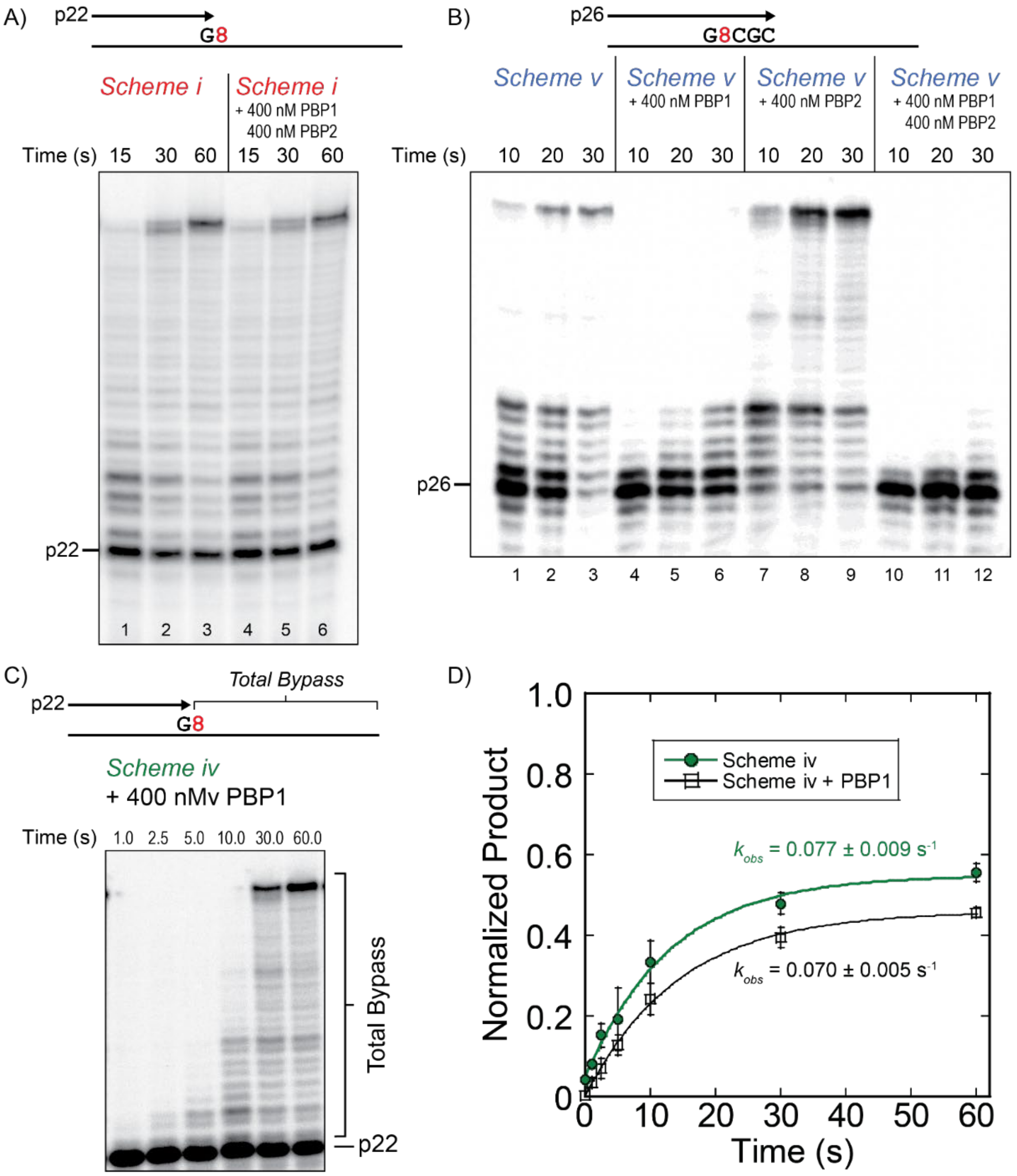

Supplemental Figure S4: PBP1 does not increase the rate of the first hand-off from a stalled PolB1 to PolY for lesion bypass. A) Pre-steady state lesion bypass kinetics were performed by *Scheme i* (containing pre-loaded PolY) on the indicated DNA substrate either alone (lanes 1-3) or in the presence of 400 nM PBP1 and PBP2 (lanes 4-6). B) Pre-steady-state extension kinetics assays were performed by *Scheme v* (containing pre-loaded PolB1; see **Fig. 6A**) on the indicated DNA substrate. Reactions were performed with PolB1 alone (lanes 1-3), or in the presence of 400 nM PBP1 (lanes 4-6), 400 nM PBP2 (lanes 7-9), or 400 nM of PBP1 and PBP2 (lanes 10-12). C) Similar reactions were performed by *Scheme iv* with 400 nM PBP1 pre-loaded in syringe A and resolved by denaturing PAGE. D) 'Total Bypass' products were quantified over time (black, -□-) and fit to **Equation 1** to obtain the observed rate constant and compared to the rate of *Scheme iv* in the absence of PBP1 (green, -●-).Error bars represent the standard deviation of three independent replicates for each time point.

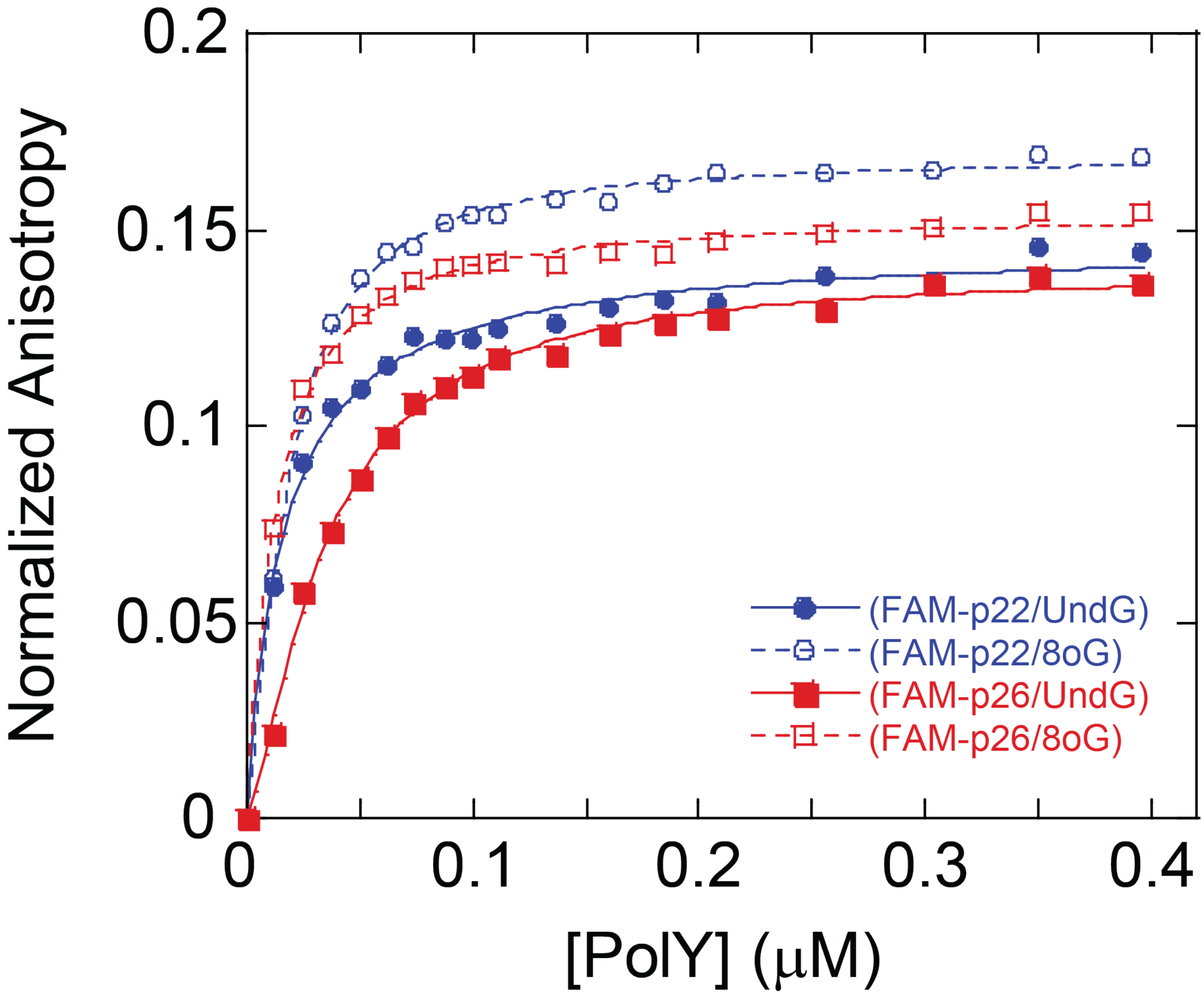

Supplemental Figure S5: Inefficient extension of PolY from the +3 intermediate is not a result of decreased DNA binding affinity. Fluorescence anisotropy was performed by titrating PolY into the indicated FAM-labelled DNA substrates. The binding curve was plotted and fit to **Equation 3** to obtain the apparent dissociation constants (K_d) for PolY binding to primers at either -1 (p22) (16.1 ± 0.4 nM) or +3 (p26) (31.2 ± 3.5 nM) from an undamaged G in the template or at -1 (25.9 ± 8.2 nM) or +3 (13.0 ± 0.4 nM) from 8-oxoG in the template.
